# The Death of Dr. F. E. Daniel, of Austin, Texas

**Published:** 1914-06

**Authors:** M. M. Smith


The Death of Dr. F. E. Daniel, of Austin, Texas.—It becomes my sad duty to announce in this issue of the *News* the death of Dr. F. E. Daniel, editor and publisher of the Texas Medical Journal; one of the dearest friends that the writer had in the medical profession, and one of the first physicians he met when he began the practice of medicine in Austin. Dr. Daniel was a man who always had time to talk to the young physician, to encourage him to push forward and forge to the front, and to offer him words of advice and comfort at a time when such advice was worth its weight in gold.

Dr. Daniel occupied a unique and distinct field in the medical profession. He stood like the towering oak in the forest, in mentality, head and shoulders above his fellows. His intellect was conspicuously one of his characteristics. His wit, repartee and sarcasm, all would shine at times. As a writer, Dr. Daniel was one of the brightest of the medical profession in the South. His reputation as an author became national.

It was the writer's high privilege to have known Dr. Daniel as few members of the profession could know him. He was truly a wonderful man. Although frail of body, yet like Alexander H. Stephens, he was possessed of a brilliant intellect.

Dr. Daniel labored more years for the upbuilding of the medical profession of Texas and he strove more earnestly and continuously to reach that goal than probably any other physician living in the State. He worked in season and out of season for legitimate medicine. His journal, "The Red Back," for many years, was the single medium, I might say, of communication among members of the profession which held the State organization together. His pen and his tongue were always ready to do battle for his profession. His labors for many, many years in the legislative halls and on the various committees of the Association were given gladly and freely; and he championed, through his publication, the new, advanced theory of eugenics, which stands for the betterment of the race and will result in more health and consequent happiness to mankind.

Dr. Daniel was one of the characteristic, old-school physicians and a typical Southern gentleman. He was high-minded about everything and could not stoop to anything low or small. He was a firm believer in the motto of Davy Crockett—"Be sure you are right, and then go ahead." He did not care whether he stood with the majority or was in the minority, once he was convinced he was right in his contentions, and he was worthy of his steel either in physical contest or in public debate.

The writer never visited Austin without paying a social call upon Dr. Daniel, and we can picture him at this moment, in his customary attitude, body erect and head well poised, always immaculately groomed, eyes sparkling with interest and face alight with animation, interspersing his remarks with appropriate gestures, as he stood to made an address in his inimitable style.

He possessed the diction of a Woodrow Wilson, the fire and and venom, if needed, of a John J. Ingalls, the force and determination of a Theodore Roosevelt and the eloquence of a William J. Bryan.

Daniel, my fellow journalist, I knew you better and perhaps understood you more thoroughly than any living physician today. I can truly say in your presence, if you are looking down upon me at this moment, that you really bore no man malice. You had made your peace with every human being and with your God. You loved mankind and every living thing on this earth. You held the highest ideals in life and constantly used your pen to persuade others to your viewpoint. Your admiration for the women of our country and for the protection of their honor was characteristic of you, and your gallantry and chivalry shone forth at all times.

Truly man in his lifetime attempts to play many roles, and yet but few become masters in more than one. But nature was partial to you and endowed you with many rare gifts and enabled you to achieve, through your wondrous talents, success in many lines. You were gifted as an orator, as a writer and as a conversationalist. You were learned not only in medicine, but also in the sciences and the arts.

As a reconteur, you showed much merit, as evidenced in your work entitled "The Recollections of a Rebel Surgeon." The scientific side of your nature revealed itself in your wonderful narrative, "The Strange Case of Dr. Bruno," while your imagery therein rivaled that of an Edgar Allan Poe. And your religion, I feel, was truly and beautifully expressed in the classic eulogy you delivered before the State Association upon the death of Dr. R. M. Swearingen.

As the loving and indulgent father, the gentle and devoted husband and indeed in every walk of the home, your example was one to be emulated.

Dear friend and co-laborer, we miss thee from our midst. Thy departure from us hath left a vacant place in our hearts which cannot be filled by another. If it be permitted those of us who are left to communicate with those who have passed a little ahead to the Great Beyond, then would we give thee this message: "Your fellow physicians all loved you. They treasure you in their memories. They miss you and would strive to accomplish those high ideals for which you so earnestly labored. They know you must be happy in the company of the great physicians who preceded you to that celestial shore, where you may commune with the gallant Swearingen, the lovable Manning, the stately Wooten, the retiring Litten, the courageous Wilson, the earnest Wallace, the kindly Gardner, the devoted Harrison and the hundreds of others who have crossed to that other side."

Send to us messages of good cheer and give inspiration to take up the burden where you laid it down and strength to move forward in our professional work, so that we may contribute our portion of the needed labor for the ultimate uplift of humanity and the purification of the human race.—Dr. M. M. Smith, *Medical News.*

